# The impact of the COVID-19 pandemic on the diagnosis and treatment of ocular cancer

**DOI:** 10.5935/0004-2749.20230023

**Published:** 2022-02-18

**Authors:** Vicente Conrado Fontes Junior, Arthur Gustavo Fernandes, Melina Correa Morales, Rubens Belfort Neto

**Affiliations:** 1 Departamento de Oftalmologia e Ciências Visuais, Escola Paulista de Medicina, Universidade Federal de São Paulo, São Paulo, SP, Brazil

**Keywords:** Ocular neoplasms, COVID-19, Social isolation, Quarantine, Neoplasias oculares, COVID-19, Isolamento social, Quarentena

## Abstract

**Purpose:**

To evaluate the impact of social isolation due to the COVID-19 pandemic on the number of new cases and therapeutic approaches at the Ocular Oncology division from the *Universidade Federal de São Paulo* (UNIFESP).

**Methods:**

A retrospective study was conducted by medical records review of new patients treated before the pandemic from March 2019 to September 2019 (pre-pandemic group) and during the pandemic from March 2020 to September 2020 (pandemic group). Data regarding age, sex, ethnicity, place of origin, clinical diagnosis, time since referral, and proposed therapy were analyzed.

**Results:**

We analyzed 186 new cases, 122 from the pre-pandemic group and 64 from the pandemic group, representing a decrease of 47.54% in new cases. There was no statistically significant change in sex, race, state of origin, history of cancer, age, or time with suspected cancer (p>0.05). A higher frequency of malignancies was observed in the pandemic group (68%) when compared to the pre-pandemic group (48.48%). Benign tumors were the most common diagnosis in the pre-pandemic group (41.80%), while conjunctival squamous cell carcinoma was the modal diagnosis in the pandemic group (31.25%). There was a decreasing trend (p=0.097) in the number of surgeries (-7.63%) and an increase in topical treatment (+10.68%). There was also a tendency to perform fewer surgeries in benign tumors and decreased follow-up visits.

**Conclusion:**

Our findings showed a significant decrease in the number of new cases referred to the Ocular Oncology service. Moreover, the pandemic led to a switch in the therapeutic approach with preference to non-invasive treatments that would demand operating rooms. A drastic increase of cases perhaps in advanced stages might be expected because of the decrease observed in the first six months of quarantine.

## Abstract (pt)

**Objetivo:**

Avaliar o impacto do isolamento social devido à pandemia COVID-19 sobre o número de casos novos e abordagens terapêuticas.

**Métodos:**

Estudo retrospectivo por revisões de prontuários de novos pacientes tratados antes da pandemia, de março de 2019 a setembro de 2019 (grupo pré-pandemia), e durante a pandemia, de março de 2020 a setembro de 2020 (grupo pandemia). Dados analisados incluíram idade, sexo, etnia, local de origem, diagnóstico clínico, tempo desde o encaminhamento e terapia proposta.

**Resultados:**

Um total de 186 novos casos foram analisados, sendo 122 do grupo pré-Pandemia e 64 do grupo Pandemia, representando uma redução de 47,54%. Não houve alteração estatisticamente signi ficativa quanto a sexo, raça, estado de origem, história do câncer, idade ou tempo de encaminhamento (p>0,05). Observou-se maior frequência de malignidades no grupo pandemia (68%) quando comparado com o grupo pré-pandemia (48,48%). Os tumores benignos foram os casos mais diagnosticados no grupo pré-pandemia (41,80%), enquanto no grupo pandemia o diagnóstico mais frequente foi o carcinoma de células escamosas de conjuntiva (31,25%). Houve tendência (p=0,097) de diminuição no número de cirurgias (-7,63%) e de aumento no tratamento tópico (+10,68%). Houve também uma tendência a diminuição de indicação cirúrgica em tumores benignos e diminuição dos retornos imediatos.

**Conclusão:**

Nossos achados mostram uma diminuição significativa no número de novos casos encaminhados ao setor de Oncologia Ocular. Além disso, a pandemia levou a uma mudança na abordagem terapêutica com preferência a tratamentos não invasivos, a fim de evitar o uso de salas de cirurgia. Um aumento drástico de casos, talvez em estágios avançados, pode ser esperado como resultado da diminuição observada nos primeiros 6 meses de quarentena.

## INTRODUCTION

In December 2019, the first cases of a respiratory failure of unknown cause were reported in Wuhan, Hubei, China. In February 2020, the World Health Orga nization named this new entity COVID-19. Caused by a type of coronavirus, the main clinical features include respiratory symptoms and fever, but fatigue, myalgia, diarrhea, among others were also noted^(1,2)^. In the following months, the disease became a global pandemic, with the first confirmed case in Brazil on February 26^th^. Ocular findings of COVID-19 have already been reported and include hyperemia, chemosis and tearing, or even as a follicular conjunctivitis^(3)^. A recent study conducted in the Federal University of São Paulo identified for the first-time retina disturbances caused by the new coronavirus, showing hyperreflective lesions at the level of the ganglionic cells layer and internal plexiform layer^(4)^. In addition to direct or indirect contact with respiratory secretions, the transmission is also possible by the ocular surface, in contact with the mucosa or tears^(5)^.

With the social distancing caused by the pandemic, it was necessary to reduce visits in health centers, followed by changes in the approach of the most diverse types of tumors. Many patients even missed chemotherapy sessions while others had surgeries postponed. Each service acted according to its own experience in an attempt to minimize the consequences of the pandemic in cancer patients^(6)^.

In Brazil, conjunctival squamous cell carcinoma and choroidal melanoma are among the most frequent ocular neoplasms in the adult population^(7)^. These are at risk of unfavorable evolution and the lack of early diagnosis and appropriate follow-up may increase the morbidity and mortality of the affected population.

In addition to the recommendation of social isolation and the fear of seeking for a health service, there are still places where the contingency of professionals is being drained to combat the new coronavirus. Thus, many cancer patients delayed the diagnosis and others lost follow-up, which can generate serious consequences in the evolution of the disease. Although several oncology units have estimates of the fall in the number of visits, few studies compared this impact before and after the pandemic.

The purpose of the current study was to evaluate the impact of social isolation due to the COVID-19 pandemic on the number of new cases and therapeutic approaches at the Ocular Oncology division from the *Universidade Federal de São Paulo* (UNIFESP).

## METHODS

We collected data from new patients evaluated in the Ocular Oncology division of UNIFESP for a period of six months; from March 16^th^ to September 16^th^, 2020, classified as pandemic group. The chosen start date was a milestone that impacted our service, when in the municipality of São Paulo-SP was declared state of emergency and social isolation measures were initiated.

Data from these six months were compared with data of new cases from the same period of the previous year, between March 16 and September 16, 2019, which corresponded to the pre-pandemic group.

Among the variables analyzed, we included: age, sex, race, family history of cancer, origin (by state), referral time (which refers to the time between the diagnosis of cancer until reception by our team), clinical diagnosis (which was subdivided into conjunctival squamous cell carcinoma - SCC, choroidal melanoma, other malignant tumors, benign tumors and other diagnoses) and therapeutic approaches (subdivided into topical treatments, surgical treatments, follow-up, or referral).

The analyses were performed in the STATA 14.0 program. Frequency tables were used for descriptive analyses. Chi-Square and Fisher’s Exact tests were applied to compare categorical variables between groups. Continuous variables were compared using the Mann-Whitney test. For all tests, statistical significance was considered when p<0.05.

## RESULTS

A total of 186 new cases were included in the current study. Participants were classified under the pre-pandemic group (n=122) and the pandemic group (n=64). There was a 47.54% reduction in the number of new cases that arrived in our service due to the Corona Virus pandemic. Table 1 shows the demographic characteristics of the participants of the 2 groups.

**Table 1:** Descriptive analysis of the sample according to the group

	Pre-Pandemic group	Pandemic group	p-value
Sex; N (%)			
Male	69 (56.56)	43 (67.19)	0.159
Female	53 (43.44)	21 (32.81)	
Race; N (%)			
White	70 (57.38)	36 (56.25)	0.352
Brown	33 (27.05)	23 (35.94)	
Black	18 (14.75)	5 (7.81)	
Yellow	1 (0.82)	0 (0.00)	
Origin (by state); N (%)			
SP	117 (95.90)	64 (100.00)	0.872
ES	2 (1.64)	0 (0.00)	
DF	1 (0.82)	0 (0.00)	
MG	1 (0.82)	0 (0.00)	
RN	1 (0.82)	0 (0.00)	
Family history of cancer; N (%)			
Yes	35 (28.69)	18 (28.22)	0.404
No	87 (71.31)	46 (71.88)	
Age; mean±std	54.96 ± 19.98	53.87 ± 20.06	0.703

There was no statistically significant difference in sex (p=0.159), race (p=0.352), state of origin (p=0.872), history of cancer (p=0.404) or age (0.703) between the two study periods. Although there was no significant difference in the state of origin, it is observed that 100% of the new cases in the pandemic period came from the state of São Paulo, where our service is located.

The referral time was 10.23 ± 16.85 weeks in the pre-pandemic group and 9.01 ± 14.11 weeks in the pandemic group, though not significantly different (p=0.757).

During the pre-pandemic period, 99 cases of tumor were confirmed with 51 cases (51.52%) of benign lesions and 48 cases (48.48%) of malign lesions. During the pandemic period, 40 cases of tumor were confirmed with 16 cases (32.00%) of benign lesions and 34 cases (68.00%) of malign lesions. The increase on the frequency of malignancies in the pandemic period was statistically significant (p=0.024).

Table 2 shows the details of diagnoses in the two periods. Considering all cases, benign tumors were the most diagnosed in the pre-pandemic group (41.80%), while in the pandemic group, the modal diagnosis was conjunctival squamous cell carcinoma (31.25%).

**Table 2:** Frequencies of diagnostic hypothesis according to the group

	Pre-Pandemic group N (%)	Pandemic group N (%)
Benign tumors	51 (41.80)	16 (25.00)
Conjunctival SCC	27 (22.13)	20 (31.25)
Choroidal melanoma	12 (9.84)	9 (14.06)
Other malignant tumors	9 (7.38)	5 (7.81)
Other diagnoses	23 (18.85)	14 (21.88)
Total	**122 (100.00)**	**64 (100.00)**

Table 3 shows the frequency of treatments proposed as first-line for each case.

**Table 3:** Frequencies of treatments according to the group

	Pre-Pandemic group N (%)	Pandemic group N (%)	p-value
Benign tumors			
Observation	29 (56.86)	7 (43.75)	0.088
Surgery	12 (23.53)	1 (6.25)	
Topical treatment	7 (13.73)	5 (31.25)	
Laser	2 (3.92)	1 (6.25)	
Referral	1 (1.96)	2 (12.50)	
Conjunctival SCC			
Topical treatment	18 (66.67)	16 (80.00)	0.567
Surgery	8 (29.63)	3 (15.00)	
Referral	1 (3.70)	1 (5.00)	
Choroidal melanoma			
Surgery	9 (75.00)	9 (100.00)	0.165
Referral	3 (25.00)	0 (0.00)	
Other malignant tumors			
Surgery	5 (55.56)	1 (20.00)	0.238
Referral	4 (44.44)	4 (80.00)	
Other diagnoses			
Monitoring	10 (43.48)	7 (50.00)	0.638
Referral	9 (39.13)	7 (50.00)	
Surgery	2 (8.70)	0 (0.00)	
Topical treatment	2 (8.70)	0 (0.00)	
Total	**122 (100.00)**	**64 (100.00)**	

There were no statistically significant differences in the management for benign tumors (p=0.088), conjunctival SCC (p=0.567), choroidal melanoma (p=0.165), other malignant tumors (p=0.238) or other diagnoses (p=0.638). However, there was a decreasing trend (p=0.097) in the number of surgeries (-7.63%), and an increasing trend in topical treatment (+10.68%) and referrals (+9.58%) when compared to the same period in 2019.

Table 4 shows the frequency of return time between the first and second visits.

**Table 4:** Return time frequencies according to the group

	Pre-pandemic group N (%)	Pandemic group N (%)
Immediate	51 (41.80)	22 (34.38)
1 month	33 (27.05)	17 (26.56)
3 months	7 (5.74)	7 (10.94)
6 months	14 (11.48)	4 (6.25)
1 year	2 (1.64)	3 (4.69)
Discharged	15 (12.30)	11 (17.19)
Total	**122 (100.00)**	**64 (100.00)**

There is no statistically significant difference in relation to the return time (p=0.355), although there is a tendency to decrease immediate (in less than 1 month) return visits and increase in discharges and annual returns.

## DISCUSSION

Our findings showed a significant decrease in the number of new cases referred to our Ocular Oncology service, suggesting these cases will present with more advanced disease in the future. The fact that we did not receive cases from other states during the pandemic period allows us to speculate that the delay in diagnosis will be even greater in regions that have a more difficult access to health care. In this context, the discussion about the use of resources such as telemedicine that can facilitate diagnosis and treatment options by health professionals who are in remote regions is very important. During the first months, we screened patients through telephone for the need to show up for an in-person evaluation. This also enabled better communication among physicians, facilitated the assessment of the real need for referral of certain cases and assisted in the therapeutic approach.

The increase in the proportion of malignancies in the pandemic group could indicate that only the more classic presentations were referred, with suspected cases of benign lesions being observed, leaving room for suspected cases of malignant tumors. Although the most appropriate conduct in a pandemic with social distancing, it carries the risk of errors in diagnosis leading to a delay in appropriate therapy of malignant cases.

Although not statistically significant, we observed a reduction in the number of surgeries performed in cases with benign tumors. This was a trend observed in several areas of medicine, opting for a therapeutic approach with preference for noninvasive treatment, saving the limited operating room time to malignant cases and holding elective cases, a trend seen worldwide^(8)^.

Several recommendations have been made in order to keep surgical procedures safe^(9)^. In ophthalmological procedures, it was observed, through simulated surgeries, that the best way to avoid contamination with respiratory droplets was the use of a mask by the patient, in addition to a surgical field with insulating adhesive on the entire surface^(10)^. The decision on the postponement of each procedure should be individualized, considering not only the conditions in which the surgery will be performed, but also the urgency of the case and state of the pandemic of the population in question.

Even with a longer interval for the follow-up visit of patients with benign lesions during the pandemic, we observed similar time of visits compared to the pre-pandemic group. This is probably due to the higher proportion of patients diagnosed with malignant diseases in the pandemic group, requiring earlier visits.

A drastic increase in cases, perhaps in advanced stages, can be expected as a result of the decrease in the number of cases observed in the first six months of quarantine. Therefore, public health authorities, institutions, and professionals who deal with these patients must be prepared to meet up this accumulated demand. The reflexes of the COVID-19 pandemic in public health go far beyond those affected by this new disease and generate consequences in the most diverse areas of medicine.

In conclusion, the number of new cases referred to our service during the pandemic was significantly lower when compared to the same period from the previous year. The pandemic also led to a switch in the therapeutic approach with preference to non-invasive treatment techniques that would not require operating rooms. One could expect an increase of cases in more advanced stages to present in the near future.
